# The prevalence of triggers in paediatric migraine: a questionnaire study in 102 children and adolescents

**DOI:** 10.1007/s10194-011-0397-2

**Published:** 2011-11-01

**Authors:** Dorothée Neut, Antoine Fily, Jean-Christophe Cuvellier, Louis Vallée

**Affiliations:** 1 Division of Pediatric Neurology, Department of Pediatrics, Lille Faculty of Medicine and Children’s Hospital, Lille, France; 2 Service de Médecine Néonatale, Hôpital Jeanne de Flandre, avenue Eugène Avinée, Lille cedex, France

**Keywords:** Migraine, Trigger factors, Child, Adolescent

## Abstract

The prevalence and characterization of migraine triggers have not been rigorously studied in children and adolescents. Using a questionnaire, we retrospectively studied the prevalence of 15 predefined trigger factors in a clinic-based population. In 102 children and adolescents fulfilling the Second Edition of The International Headache Classification criteria for paediatric migraine, at least one migraine trigger was reported by the patient and/or was the parents’ interpretation in 100% of patients. The mean number of migraine triggers reported per subject was 7. Mean time elapsed between exposure to a trigger factor and attack onset was comprised between 0 and 3 h in 88 patients (86%). The most common individual trigger was stress (75.5% of patients), followed by lack of sleep (69.6%), warm climate (68.6%) and video games (64.7%). Stress was also the most frequently reported migraine trigger always associated with attacks (24.5%). In conclusion, trigger factors were frequently reported by children and adolescents with migraine and stress was the most frequent.

## Introduction

‘Triggers are factors that, alone or in combination, induce headache in susceptible individuals’ [1]. They are important in migraine management since their avoidance may result in a better control of the disorder. In spite of their importance, literature on triggers for childhood migraine is sparse [2, 3] and less is known regarding the influence of gender, migraine subtype, or attack frequency on the distribution of triggers.

The aim of the present study was (1) to assess the prevalence of 15 predefined trigger factors (TF) that have been reported both in adults and paediatric patients using a telephone questionnaire in an unselected clinic-based population of children and adolescents, (2) to assess the influence of gender, migraine subtype, and attack frequency on the distribution of TF, and (3) to evaluate the likelihood of TF to precipitate an attack, using a 4-point scale (rarely, often, very often or always).

## Patients and methods

The study population consisted of children and adolescents suffering from migraine according to the Second Edition of The International Headache Classification (ICHD-II) criteria. These patients were drawn from the database of one of the authors (JCC). This author includes in this database all paediatric patients seen for headache in clinical practice at the neuropaediatric outpatient department of a tertiary care teaching hospital (Lille University Hospital Center) since March 2003. Patients were randomly selected. All evaluations were conducted through phone by the same person (DN). The interviewed person was one of the child/adolescent’s parents, and as far as possible, the child or adolescent was personally interviewed. Eligible subjects were children and adolescents (<17 years) who fulfilled the ICHD-II criteria for paediatric migraine with and/or without aura at the time of study, experienced migraine attacks for at least 6 months before study entry, and had had less than 15 headache days per month during last 3 months. Patients who had chronic daily headache were excluded as it was felt that this group may not readily reflect changes induced by potential triggers.

The first part of the questionnaire addressed demographic factors (age, gender) and present migraine characteristics. Migraine-related variables were: migraine subtype (migraine without and/or with aura according to the ICHD-II criteria), mean attack frequency per month in the last 6 months, and preventive treatment, whether pharmacological or behavioural. The second part of the questionnaire addressed 15 possible trigger factors that were included based on reports in the paediatric and adult literature. This predetermined list of TF included behavioural, dietary, environmental, infectious, traumatic, hormonal factors, and other. The participants had to state for each of these 15 TF whether it precipitated headache in their own personal experience using a 5-point scale depending on their likelihood to precipitate an attack of MA or MO (0 = never, 1 = rarely (>0 to <1/3 attacks), 2 = often (1/3 to <2/3 attacks), 3 = very often (2/3 to <1 attacks) or 4 = always) and they had to specify the time elapsed between exposure to TF and attack onset, answers being categorised as <3 h, 3 to <6 h, 6 to <12 h, and 12–24 h. It was possible to add additional TF not included in this list and answer the questions as well. Since patient care was not altered by inclusion in the study, ethics committee approval was not necessary but all data were kept confidential. All analyses were performed using Stata software (7.0 version, CDC, Atlanta, USA). Percentages were rounded to a whole. *p* values <0.05 were considered as statistically significant. Chi squared tests were used to evaluate risk factors prevalence according to sex, age, migraine subtype and frequency of attacks.

## Results

Data collection was made between 15 February 2010, and 30 April 2010. Of the 128 subjects with a diagnosis of migraine included in the database that were contacted by phone, 102 actually fulfilled the inclusion criteria. All agreed to answer the questionnaire. The interviewed person was one of the parents in 43.2% (*n* = 44), the child in 25.5% (*n* = 26), and both parent and child in 31.4% (*n* = 32). There were 55 boys (54%) and 47 girls. Mean age of the subjects was 12 years (7–16). Fifty-three (52%) patients were in the 7–12 years range, 49 (48%) in the 13–16 years range. Seventy-one patients had migraine without aura (69%), 22 migraine with aura (22%), and 9 patients both migraine without aura and migraine with aura (9%). Number of headache days per month was <1 in 19 patients (18%), 1–2 in 19 patients (18%), 3–4 in 22 patients (21%), 5–6 in 5 patients (5%), 7–8 in 6 patients (6%), 9–10 in 1 patient (1%), and 11–15 in 7 patients (7%). Five (4.9%) had behavioural preventive therapy and five (4.9%) had pharmacological preventive therapy*.* Preventive medication was amitriptyline (*n* = 2), dihydroergotamine (*n* = 1), flunarizine (*n* = 1) and topiramate (*n* = 1).

**Fig. 1:**
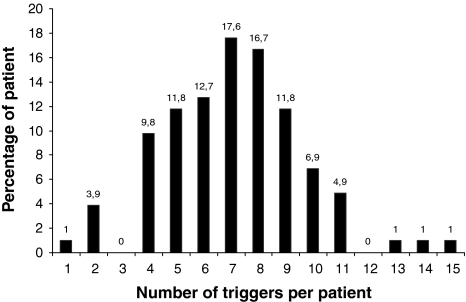
Percentage of patients according to the number of trigger factors

**Fig. 2:**
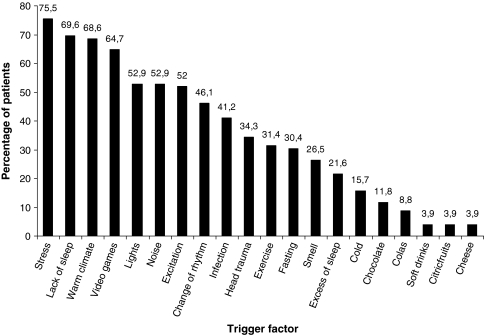
Percentage of patients reporting each individual trigger factor

All patients reported at least one TF. The mean number of TF reported per subject was 7 (median 7) and the maximum number of TF reported per subject was 15 (Fig. 1). Stress was the most frequently reported TF (75.5%), followed by the lack of sleep (69.6%), warm climate (68.6%) and video games (64.7%) (Fig. 2). A total of 70.6% (*n* = 72) of patients reporting TF reported at least one TF that very often precipitated an attack of migraine and 62.7% (*n* = 64) reported a TF that always precipitated an attack. Stress was the most frequently reported TF always associated with attacks (24.5%) whereas warm climate was the most frequently reported TF often or very often associated with attacks (24%). Other TF not listed on the questionnaire were mentioned by the patients and/or their parents: air conditioning, car journeys, enclosed atmosphere, cigarettes smoke, warm and humid climate, wind, prolonged reading, concentration with visual fixation, and dietary TF such as eggs, sweets, or fat food.

**Fig. 3:**
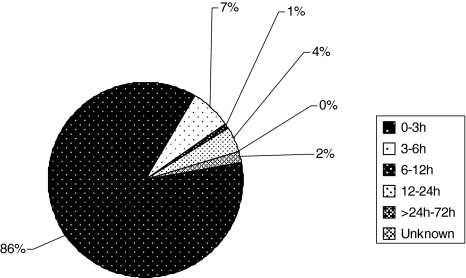
Mean time elapsed between exposure to a trigger factor and attack onset

Figure 3 shows mean time elapsed between exposure to a TF and attack onset. It was comprised between 0 and 3 h in 88 patients (86%). It was also comprised between 0 and 3 h in 40 (85%) of the 47 patients reporting stress as a factor triggering always or very often an attack.

### Correlations and group comparisons

When considering demographic (age, gender) and clinical (frequency of attacks, type of migraine) features, some differences between groups were statistically significant. Missing a meal was more often mentioned as a TF in the 7–12 years range (45% vs. 14% in the 13–16 years range, *p* = 0.001). Conversely, odours were more often mentioned as a TF in the 13–16 years range (41 vs. 13% in the 7–12 years range, *p* = 0.002). Warm climate and agitation/excitation were reported as a TF by respectively 78 and 64% of boys versus respectively 55% (*p* = 0.014) and 38% of girls (*p* = 0.011). When considering attacks frequency, stress and odours were reported as a TF by respectively 88 and 41% of children with more than 2 AM per month versus respectively 67% (*p* = 0.018) and 41% (*p* = 0.005) of those with less than 2 AM per month. Odours, chocolate and mild head trauma were more often mentioned as a TF in patients with migraine with aura (respectively 42, 23 and 48% of patients) than in patients with migraine without aura [respectively 20% (*p* = 0.019), 7% (*p* = 0.025) and 28% (*p* = 0.048) of patients].

## Discussion

Few papers have been dedicated to the study of TF for childhood migraine [2–4]. All children and adolescents in our study had at least one TF. In the literature, between 75 and 89% of adult migraineurs have at least one TF [5–8]. In the study by Chakravarty et al. [3], one or more triggers could be detected in 94% of children when studied retrospectively and 100% of them when studied prospectively. The mean number of TF reported per patient [7] in our study was comparable to the adult studies of Kelman (6.7) and Theeler (8.9) [5, 8].

The most common individual trigger was stress which was reported by 75.5% of patients, a figure close to that was found by Chakravarty et al. (78%) [3]. We can but agree with Carod-Artal et al. [9] when they wondered that stress was a TF in different societies, in spite of cultural differences between them. The study by Chakravarty et al. [3], which seems to be the only study specifically devoted to paediatric migraine TF as a whole, available so far, was conducted in India. Climatic, ethnic, dietary and socio-cultural factors are very different in India from those in our country. In adult studies, stress is experienced as a major precipitant of migraine and is reported as a TF by 59–79% of patients [5–7]. Of note, stress always triggered an attack in 24.5% of patients. We did not distinguish between occurrence during stress and occurrence after stress, adult studies having shown that the former is more often associated with the triggering of an attack than the latter [10]. One may also suspect a confusion bias between lifestyle changes and stress. For example, the start of the new school year may account as a TF as both a lifestyle change and a stress *per se*. In the Thai study by Visudtibhan et al. [11], some patients had preferentially attacks during the first days of holidays. This may be due to lowering of school pressure or to relaxation after school stress.

Lack of sleep was reported by 69.6% of patients and was generally put aside with fatigue. Late onset of sleep was reported as an occasional TF by 32% of patients in the series of Kelman, whereas 44 to 57% of adult migraineurs report lack of sleep as a TF [5, 6]. Excess of sleep was cited by 21.6% of children and adolescents. Bruni et al. [12] have shown that enhancing sleep quality resulted in the reduction of migraine and tension type headache frequency.

Hot and cold weather were reported by 68.6 and 15.7% of patients, respectively. In adults, climate is cited by 53.2–71% of patients [5, 13]. Hot weather was the main TF triggering an attack “very often” or “often” in our study. In the paediatric study by Chakravarty et al. [3], hot humid weather was a TF in 94% of patients. This study was conducted in Eastern Indian, whose climate is very different from North of France, which is generally mild and rainy. Moreover, there are few days with hot weather during a typical year in North of France. Mukamal et al. [14] have shown that a transient rise of ambient temperature increased the risk of headache requiring emergency department evaluation, with approximate 7.5% higher risk for each 5°C increment in temperature. In a paediatric prospective study using electronic momentary assessment methodology, relative humidity and presence of precipitation were significantly predictive of new headache onset [4]. By contrast, no child reported humidity and rainy days as a TF in our study.

Bright lights were reported by 52.9% of patients, a figure similar to adult literature data (29–61%) [3, 5, 6, 13, 15, 16]. We cannot exclude a possible confusion between warmth, sun exposure and bright lights. In fact, it was difficult during interview to precise what the TF was during hot weather, as patients were concomitantly exposed to these three TF and one cannot exclude that several of TF might summate to trigger the headache.

Dietary precipitants have been much stressed in western literature but were rarely reported by our patients [2, 15]. Chocolate was reported by 11.8% and cheese by 3.9%. This difference between theoretical knowledge and personal experience has been studied in adults by Wöber et al. [17]. They found that, whereas chocolate and cheese were suspected by more than half of adult patients, only 15% of them reported them as real TF. On the other hand, studies on food as a trigger of migraine attacks have given insights on biochemical mechanisms involved in the genesis of the attack. Chocolate contains phenylalanine and cheese tyramine; both have vasoconstrictive properties and may initiate a headache by alteration of cerebral blood flow and release of norepinephrine from sympathetic nerve cells [2].

Triggers are not universal. Moreover, the presence of a TF does not always precipitate an attack in the same individual. Lambert and Zagami [18] hypothesised that most triggers would excite cortical neurons, leading to an inhibitition in the periaqueductal grey matter and the nucleus raphe magnus. These two brainstem nuclei would then release their ongoing descending control of dura matter sensation in the trigeminal nucleus and cause normal sensory traffic from the dura to be perceived as migraine pain. Other hypotheses argue that TF may induce the onset of cortical spreading depression in a preexisting hyper-excitable cortex of a migraine brain, initiating the process of pain generation [19]. Other authors have speculated that common migraine triggers such as stress, skipping a meal, and fatigue may promote the headache through the activation of distinct but converging neuronal pathways that would originate in several brain nuclei that respond to the most common migraine triggers. These include the lateral hypothalamus and perifornical areas that become activated during food and sleep deprivation, and the bed nucleus of stria terminalis and paraventricular hypothalamic nucleus that are involved in regulating stress response. The neuronal outputs from these nuclei converge onto the superior salivatory nucleus, which further projects to the parasympathetic sphenopalatine ganglion [20]. Indirect activation of the sphenopalatine ganglion by triggering factors would result in local meningeal release of parasympathetic-driven vasoactive and algesic mediators such as nitric oxide and acetylcholine that in turn could enhance directly or indirectly the responsiveness of meningeal nociceptors [21].

In the present study 70.6% of patients reporting trigger factors indicated at least one trigger factor that very often or always precipitated an attack of migraine. Suggesting that avoidance of these specific factors could lead to a substantial reduction of attacks is a classical tenet of paediatric migraine management, but has been recently challenged by Martin, that advised instead that clinicians may “need to approach managing headache triggers with more flexibility than simply counselling avoidance of all triggers—think ‘coping with triggers’.” [22].

Limitations of the study are the relatively small number of subjects, the tertiary care recruitment, the retrospective design and the fact that the interviews were performed by phone. In adults, it has been hypothesised that patients with headache report more stress on retrospective self-reports than do headache-free controls because of a tendency to selectively recall more stressful events on retrospective measures [23]. Thus one may consider the possibility of confounded results, due to selective memory biases in the recollection of stressful events. To our knowledge, no paediatric headache study has addressed this issue so far. There might also be an overlap between premonitory symptoms and trigger factors in migraine. It could be that mental stress trigger a migraine attack or that patients perceive more mental stress because they are in the premonitory phase of a migraine attack. This seems very unlikely as premonitory symptoms and trigger factors are very different in children, but difficulties might arise for stress and sleep problems which were reported as premonitory symptoms in respectively 13 and 2% of paediatric migraineurs in a previous study [24].

Strengths of this study are the strict headache diagnosis according to the ICHD-II criteria and the differentiation between consistent and occasional trigger factors. The study also gives data regarding the influence of gender, migraine subtype, or attack frequency on the distribution of triggers.

In conclusion, our study indicates that all children and adolescents with migraine reported at least one trigger factor and that it very often or always precipitated an attack of migraine in 70.6% of them. The most common individual trigger was stress. Of note, mean time elapsed between exposure to a trigger factor and attack onset was inferior to 3 h in most patients. These preliminary data on trigger factors for paediatric migraine need to be assessed using prospective methods, eventually using electronic diary systems, but studying trigger factors for paediatric migraine as a whole appears cumbersome and, to our knowledge, has not even done so far in adult studies.
